# A Comprehensive Atlas of E3 Ubiquitin Ligase Mutations in Neurological Disorders

**DOI:** 10.3389/fgene.2018.00029

**Published:** 2018-02-14

**Authors:** Arlene J. George, Yarely C. Hoffiz, Antoinette J. Charles, Ying Zhu, Angela M. Mabb

**Affiliations:** ^1^Neuroscience Institute, Georgia State University, Atlanta, GA, United States; ^2^Creative Media Industries Institute & Department of Computer Science, Georgia State University, Atlanta, GA, United States

**Keywords:** ubiquitin, neurological, rare diseases, angelman syndrome, transgenic

## Abstract

Protein ubiquitination is a posttranslational modification that plays an integral part in mediating diverse cellular functions. The process of protein ubiquitination requires an enzymatic cascade that consists of a ubiquitin activating enzyme (E1), ubiquitin conjugating enzyme (E2) and an E3 ubiquitin ligase (E3). There are an estimated 600–700 E3 ligase genes representing ~5% of the human genome. Not surprisingly, mutations in E3 ligase genes have been observed in multiple neurological conditions. We constructed a comprehensive atlas of disrupted E3 ligase genes in common (CND) and rare neurological diseases (RND). Of the predicted and known human E3 ligase genes, we found ~13% were mutated in a neurological disorder with 83 total genes representing 70 different types of neurological diseases. Of the E3 ligase genes identified, 51 were associated with an RND. Here, we provide an updated list of neurological disorders associated with E3 ligase gene disruption. We further highlight research in these neurological disorders and discuss the advanced technologies used to support these findings.

## Introduction

Protein ubiquitination is a posttranslational modification that involves the covalent tethering of a small 76 amino acid protein called ubiquitin to target proteins (Hershko and Ciechanover, 1998). Ubiquitination mediates many cellular functions, which include signal transduction and the removal of proteins by the ubiquitin proteasome system (UPS) (Hershko, 1996). The initiation of protein ubiquitination typically requires an ATP-dependent enzymatic cascade that is initiated with the priming of a ubiquitin onto a ubiquitin activating enzyme (E1) and the transfer to a ubiquitin conjugating enzyme (E2) (Komander and Rape, 2012; Zheng and Shabek, 2017). Ubiquitin is then covalently attached to a lysine residue on the target protein by an E3 ubiquitin ligase (E3) and this process can be repeated to create a series of ubiquitin chains (Hershko and Ciechanover, 1998). Ubiquitin chains can take various forms in length and configuration. The fate of these chains leads to multiple cellular functions, one of which provides a signal for the protein to undergo degradation by the UPS (Swatek and Komander, 2016; Yau and Rape, 2016).

Although there are only 2 E1 and 30-50 E2 genes, there are over 600 human E3 ligase genes whose diversity is accounted for by three different types of catalytic domains: Really Interesting New Gene (RING), Homologous to E6-AP Carboxyl Terminus (HECT), or Ring-Between-Ring (RBR) (Zheng and Shabek, 2017). While both RING and HECT E3 ligases transfer ubiquitin to a lysine residue on the substrate, RING E3s act as a platform to allow direct transfer of ubiquitin from the E2 to the substrate (Riley et al., 2013). HECT E3s on the other hand, contain a catalytic cysteine residue that can form a thioester bond directly with ubiquitin. The RBR acts as a hybrid protein of 2 domains, RING and HECT, with each family having various domains leading to the ubiquitination of numerous substrates (Marín et al., 2004; Zheng and Shabek, 2017). Proteins that are part of the RBR family have both a canonical RING domain as well as a catalytic cysteine residue similar to the HECT domain (Riley et al., 2013).

**Figure 1:**
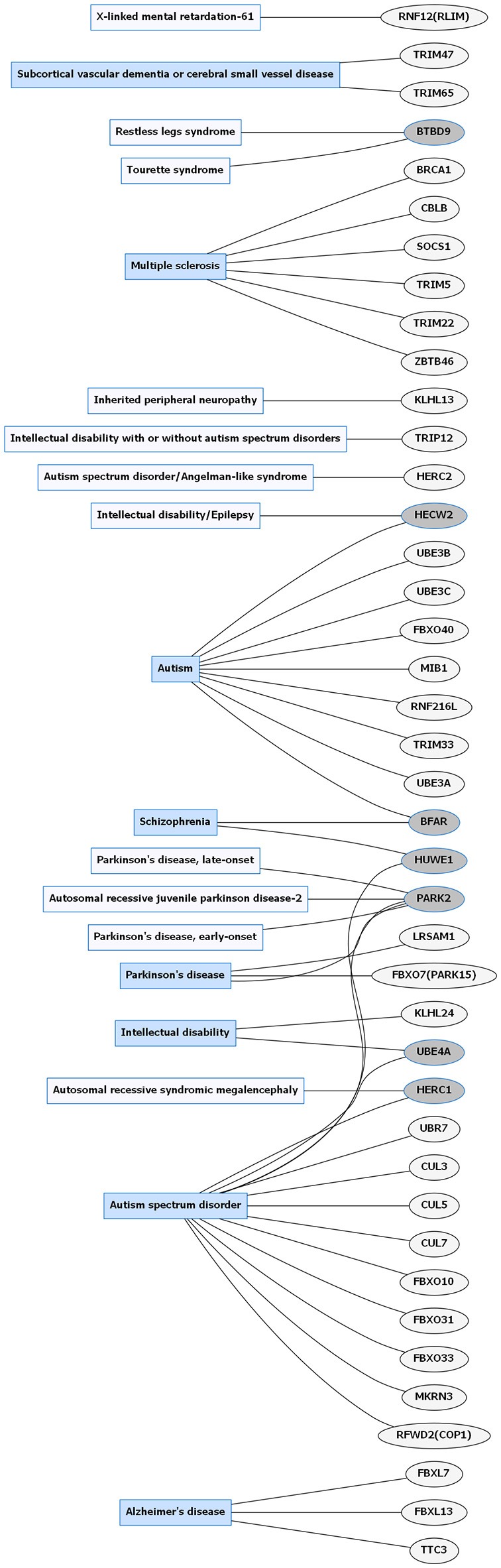
Common neurological disorders (CNDs) and E3 ligase gene associations. Diagram of CNDs correlated with E3 ligase genes that are mutated in specific disorders. Diseases shaded in blue indicate multiple genes linked to that disorder. Genes highlighted in dark gray are shared between several diseases. Figures were generated using Graphviz (www.graphviz.org).

**Figure 2:**
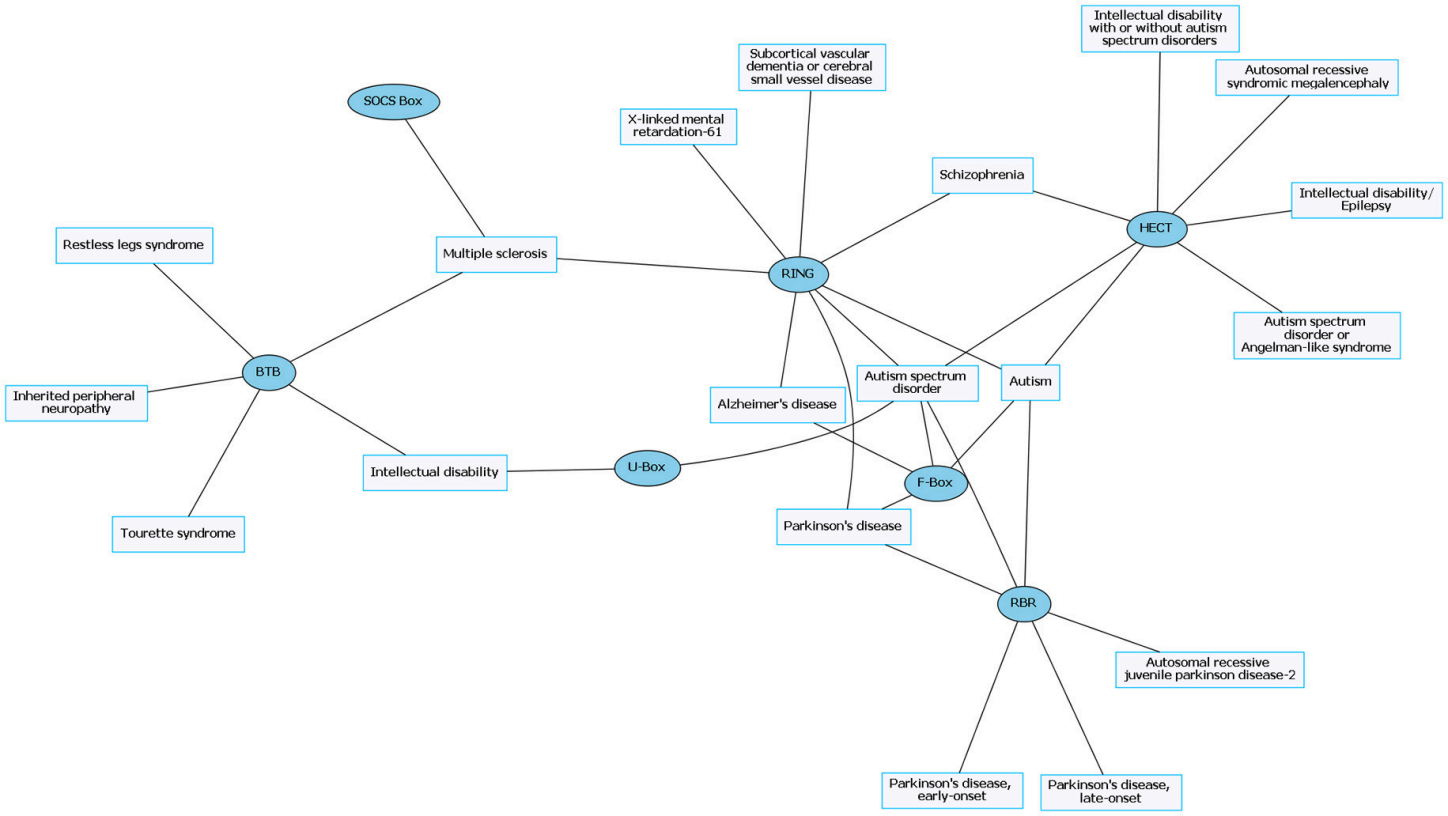
Common neurological disorders (CNDs) and their associated E3 ligase domain type. CNDs that contain mutations in E3 ligase domains (blue). Diseases such as schizophrenia and Alzheimer's disease accompany mutations in different types of E3 ligases. Figures were generated using Graphviz (www.graphviz.org).

**Figure 3:**
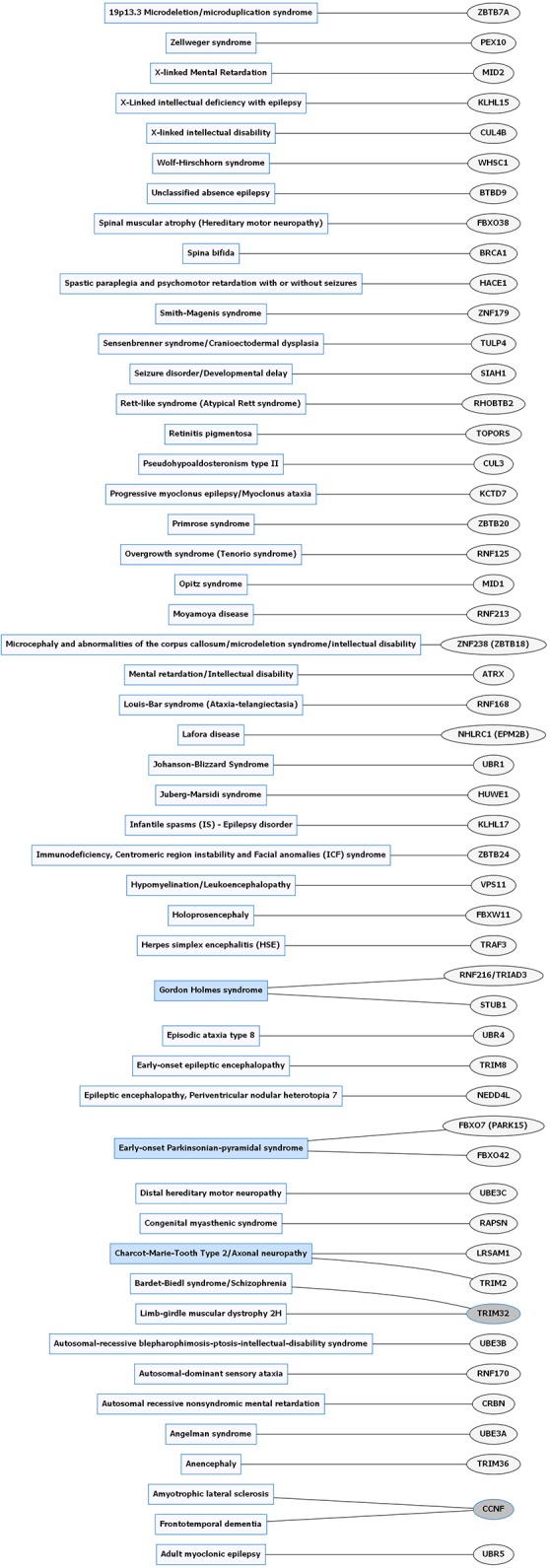
Rare neurological disorders (RNDs) and E3 ligase gene associations. Diagram of RNDs correlated with E3 ligase genes that are mutated in specific disorders. Diseases shaded in blue indicate multiple genes linked to that disorder. Genes highlighted in dark gray are shared between several diseases. Figures were generated using Graphviz (www.graphviz.org).

**Figure 4:**
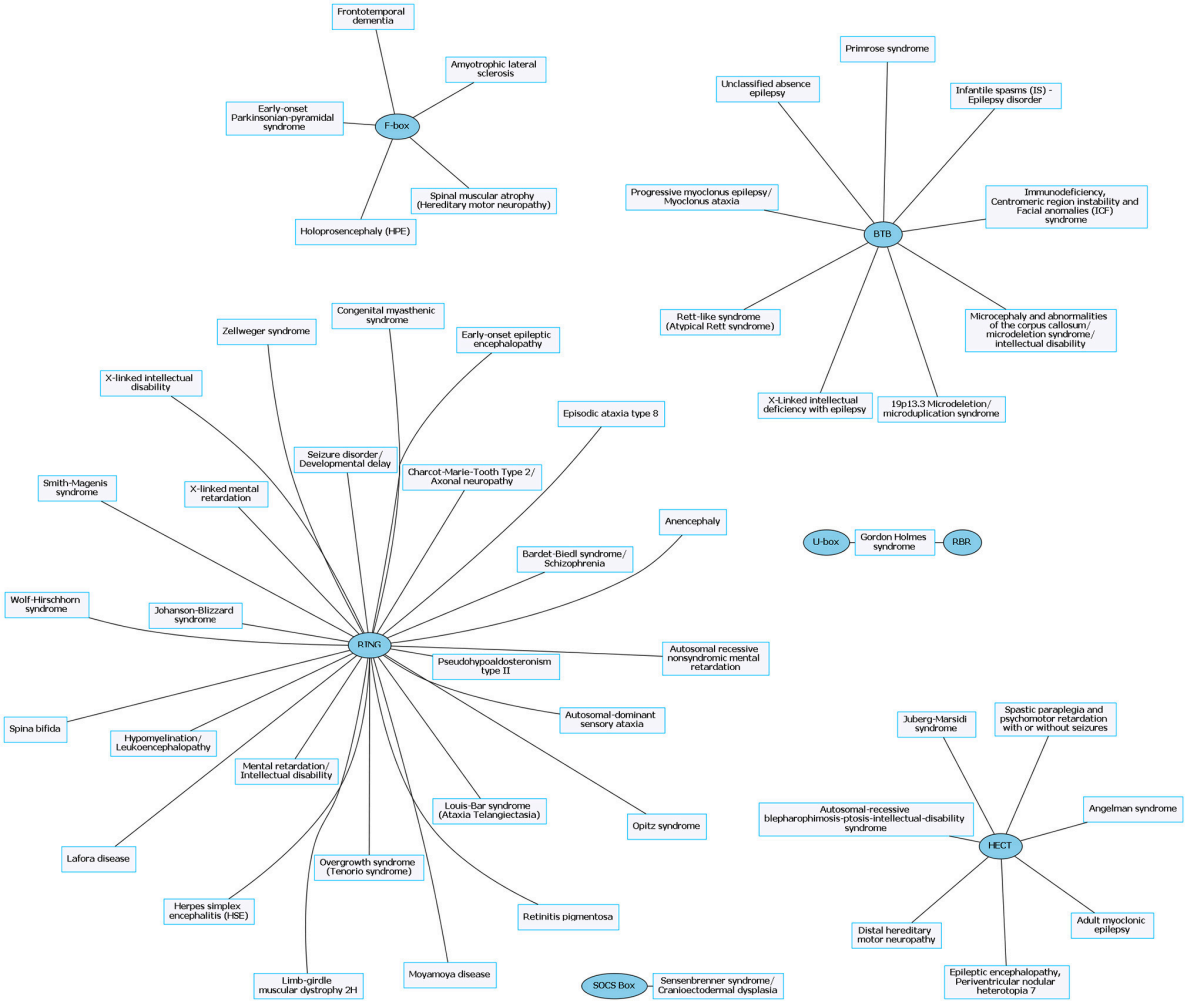
Rare neurological disorders (RNDs) and their associated E3 ligase domain type. RNDs that contain mutations in E3 ligase domains (blue). RING domain-containing E3 ligases account for ~53% of known mutated E3 ligase genes in RNDs. Figures were generated using Graphviz (www.graphviz.org).

E3 ligases have been linked to neurological disorders that include neurodegeneration, neurodevelopmental disorders, and intellectual disability (Hegde and Upadhya, 2011; Upadhyay et al., 2017), many of which have no known effective therapies. Neurological disorders are a heterogeneous group of disorders that result from the impairment of the central and peripheral nervous system, affect 1 in 6 individuals, and contribute to 12% of total deaths worldwide (WHO, 2006). Rare neurological disorders (RNDs) are a subtype of neurological diseases that represent 50% of all rare diseases, affecting fewer than 200,000 people in the United States, and are often overlooked due to lack of understanding their potential causative factors (Han et al., 2014; Jiang et al., 2014; NCATS, 2016). Although neurological disorders encompass a large array of genetic defects, next-generation sequencing (NGS) has enabled researchers to identify constituents of the ubiquitin pathway, namely E3 ligases, as causative factors for neurological disease (Krystal and State, 2014; McCarroll et al., 2014; Brown and Meloche, 2016). We speculated that recent advances in NGS resulted in a massive expansion of the list of E3 ligases mutated in neurological disease. To test this, we performed an unbiased manual database search of ~660 predicted and known human E3 ligase genes specifically mutated in neurological disease (Li et al., 2008; Hou et al., 2012). Strikingly, we found ~13% of E3 ligase genes were mutated in a neurological disorder with 83 total genes representing 70 different types of neurological diseases (Supplementary Tables 1, 2). Of the E3 ligase genes identified, 19 were associated with a CND (Figures 1, 2 and Supplementary Table 1), while 51 were associated with an RND (Figures 3, 4 and Supplementary Table 2). Thus, understanding how E3 ligase disruption is a causative factor for neurological disease may contribute to a strategy for therapeutic interventions for both CNDs and especially RNDs (Upadhyay et al., 2017).

The critical role of E3 ligases in neuropathology has been well documented in CNDs and is the result of both classic methodologies and innovative technologies that parsed out the consequences of E3 ligase disruption. However, very little is known about E3 ligase functions in RNDs such as identification of E3 ligase substrates, their definitive mechanisms, their long-term effects on a cellular and systematic level, and how to ameliorate these effects (Mabb and Ehlers, 2010; Atkin and Paulson, 2014). In the nervous system, E3 ligases are an integral part of the ubiquitin proteasome pathway involved in the turnover of proteins (Tai and Schuman, 2008; Mabb and Ehlers, 2010; Yamada et al., 2013). They localize to multiple cellular regions, which include the Golgi apparatus, centrosome, nucleus, cytoskeleton and synapse (Yamada et al., 2013). Indeed, disruptions in ubiquitin pathway components have been identified in numerous human disorders which include those related to generalized inflammation and cancer (Hegde and Upadhya, 2011; Upadhyay et al., 2017). Notably, ubiquitination mediates many forms of synaptic plasticity, which ultimately affect learning and memory (Mabb and Ehlers, 2010; Hegde et al., 2014). Below, we discuss how advanced technologies in CNDs and RNDs have been used to broaden the understanding of E3 ligases in neurological disease and have allowed researchers to exploit avenues for effective therapies. Although this review cannot encompass a thorough analysis of each disorder, we will focus on the technologies that were used to study E3 ligase disruptions in RNDs, highlighting their importance in increasing our generalized understanding of rare disease.

## E3 ubiquitin ligases and common neurological disease

### Parkinson disease

Parkinson disease (PD) is one of the most well-studied neurological diseases related to E3 ligase dysfunction. PD is characterized by dystonia, rigidity, tremors, hyperreflexia, bradykinesia, postural instability, substantia nigra gliosis and dopamine depletion, and Lewy body dementia (Halliday et al., 2014; Biundo et al., 2016). The prevalence of this disease occurs in 0.3% of the general population in the United States and 0.1–0.2% in European countries with increasing rates that occur with aging (Kowal et al., 2013; Tysnes and Storstein, 2017). Genetic mutations in the E3 ligases *LRSAM1, FBXO7* (*PARK 15*), and *PARK2* (Figure 1 and Supplementary Table 1) have been identified in Parkinson disease (Wu et al., 2005; Choi et al., 2008; Lohmann et al., 2015; Aerts et al., 2016). *PARKIN/PARK2*, belongs to the RBR family of E3 ligases, and mutations in this gene occurs in 50% of familial cases and 10–20% in sporadic cases with high penetrance in early-onset PD (Lill, 2016; Zhang et al., 2016).

A series of advanced technologies have elucidated the functional consequences of deletion and missense mutations in the *PARKIN* gene (Wu et al., 2005; Choi et al., 2008) and their use has increased the capacity for PD therapeutics (Hattori et al., 1998; Kitada et al., 1998; Hedrich et al., 2001). For example, proteomic profiling demonstrated PARKIN's role in mitochondrial autophagy (Narendra and Youle, 2011). These findings were further supported by liquid chromatography–mass spectrometry and ubiquitin Absolute Quantification of ubiquitin (UB-AQUA) proteomics. UB-AQUA is a mass spectrometry-based method that uses internal standard peptides that are isotopically labeled to quantify peptides from digested mono- and poly-ubiquitinated chains attached on substrates (Kirkpatrick et al., 2006; Phu et al., 2011). UB-AQUA was used to identify and quantify subtypes of mitochondrial ubiquitin chain linkages. Using this method, PTEN-induced putative kinase 1 (PINK1) was found to phosphorylate PARKIN leading to its activation and formation of canonical and non-canonical ubiquitin chains on mitochondria, which were PARKIN-dependent (Ordureau et al., 2014). This served as a feed-forward mechanism to promote PARKIN recruitment and mitochondrial ubiquitination in response to mitochondrial damage. Collectively, these findings were critical to understanding potential causative factors leading to PD pathogenesis (Ordureau et al., 2014).

The impairment of mitochondria in PD due to deletions of mitochondrial DNA (mtDNA) leads to respiratory-chain deficiencies especially in dopamine (DA) neurons of the substantia nigra (Bender et al., 2006). To study the effects of mtDNA deletions and other genetic mutations, conditional knock-out or knock-in mouse models using CRE recombinase and *loxP* sites were used (Soriano, 1999). The CRE-*loxP* system allows for conditional loss-of-function or gain-of-function studies in specific tissues and circumvents early life lethality and unwanted phenotypes in later stages of life by controlling when genes are expressed temporally and spatially (Sauer, 1998). A MitoPark reporter mouse line was created first by inserting a *loxP*-flanked stop-cassette upstream of the mitochondrial targeting presequence lox and yellow fluorescent protein (YFP) transgene to target it to the mitochondrial matrix. The presence of the stop cassette limits YFP expression in specific cells and is only expressed when the stop cassette is excised out with *CRE*.

Using the MitoPark mouse model, the consequences of respiratory chain dysfunction on the properties of mitochondria and DA neurons were examined after DA neuron-specific knockout of the *mitochondrial transcription factor A* (*TFAM*) (Sterky et al., 2011). In order to study the DA neurons, ROSA26^+/SmY^ mice were crossed with dopamine transporter (DAT)-*CRE* mice that express CRE under a DA transporter locus, so the offspring from these parents express YFP precisely in the mitochondria of DA neurons in the midbrain (Sterky et al., 2011). Using this model, Sterky et al. demonstrated increased fragmented and aggregated mitochondria in aged PARKIN knockout mice (Sterky et al., 2011). Specifically, striatal DA neurons displayed a reduction in mitochondria and tyrosine hydroxylase density. Surprisingly, *PARKIN* knockout MitoPark mice presented no difference in morphology or number of mitochondria with or without *PARKIN*. There was also no indication of PARKIN recruitment to defective mitochondria suggesting PARKIN did not have an effect on the progression of neurodegeneration in PD (Sterky et al., 2011).

Although limitations exist in using the PD mouse model, there have been advancements in studying the role of PARKIN in PD in a pig model. Large animals such as pigs serve as great models to study pathological phenotypes for human neurological diseases due to their physiological similarity to humans (Prather et al., 2013). Wang et al. successfully implemented the clustered regularly interspaced short palindromic repeats (CRISPR)/Cas9 system into the Bama miniature pig genome to concurrently target three distinct loci by co-injecting *cas9* mRNA and single-guide RNAs (sgRNA) which target *PARKIN, DJ-1*, and *PINK1* genes into pronuclear embryos (Wang et al., 2016). Immunofluorescence, western blotting and reverse transcription-polymerase chain reaction (RT-PCR) confirmed a significant reduction in expression of these genes compared to wild-type with a low incidence of off-target mutations via whole-genome sequencing. Despite the drawbacks to using large animals as a means of genetic modification, including the fact that it is a time-consuming and expensive procedure due to lack of embryonic stem cell (ESC) lines, the effective and specific biallelic knock-outs of genes makes this a valuable tool to study neurological disorders where other animal models have failed.

Along with studying large model organisms, the PD field has also taken advantage of using patient-derived induced pluripotent stem cells (iPSCs). IPSCs are a special type of stem cell in which human somatic cells are engineered and genetically altered to be differentiated into other types of cells in the body such as neuronal, cardiac or hepatic via distinct transcription factors (Bellin et al., 2012). IPSCs make an extraordinary model for studying human diseases because they can reveal phenotypic defects and are a renewable source. Chung et al. differentiated *PARKIN/PINK1* mutant and normal iPSC and ESC lines of midbrain DA neurons such that all cell lines demonstrated properties consistent with development of midbrain DA neurons (Chung et al., 2016). Although PARKIN and PINK1-derived iPSCs showed abnormal mitochondria (enlarged and enhanced oxidative stress), they were not prone to cell death. When given an oxidative stress inducer, carbonyl cyanide m-chlorophenyl hydrazine (CCCP), PD iPSC cell lines were more susceptible to cell death and displayed atypical neurotransmitter homeostasis (Chung et al., 2016).

In summary, upon mitochondrial depolarization, PINK1 recruits PARKIN to the mitochondrial membrane and its phosphorylation permits proteasome-dependent degradation of damaged mitochondria and enhances cell survival by suppressing apoptosis. Using whole exome genome sequencing to identify PD patients with specific PARKIN mutations that demonstrate dysfunctional mitophagy, therapeutic interventions could be targeted to prevent oxidative stress or promote regeneration of these cells via stem cell technologies specifically to DA neurons of the midbrain.

## E3 ubiquitin ligases and rare neurological disorders

Extensive examination of RNDs are difficult to accomplish due to the small number of human populations that have RNDs; however, the use of *in vitro* and *in vivo* models along with the genomic tools described above have made it possible to identify mutations of genes, particularly E3 ligases, and to recapitulate mutations observed in RNDs.

### Angelman syndrome

Angelman syndrome (AS) is a neurodevelopmental disorder that is one of the most well studied RNDs. Although specific symptoms vary in individual cases, AS is characterized by intellectual disability, developmental delay, distinct behavioral patterns such as a happy demeanor with prolonged, inappropriate laughter and smiling, speech impairment, seizures, abnormal sleep patterns, and ataxia (Williams et al., 2006; Tan and Bird, 2016). AS occurs in about one out of every 12,000 births (Steffenburg et al., 1996). Over 90% of AS cases are caused by mutations in the E3 ligase, *UBE3A* or deletions in the 15q11-13 maternal region containing *UBE3A* (Figure 3 and Supplementary Table 2; Kishino et al., 1997; Matsuura et al., 1997; Sutcliffe et al., 1997). Similar to *UBE3A*, mutations in another E3 ligase, *HERC2*, also lead to AS-like phenotypes (Puffenberger et al., 2012).

**Figure 5:**
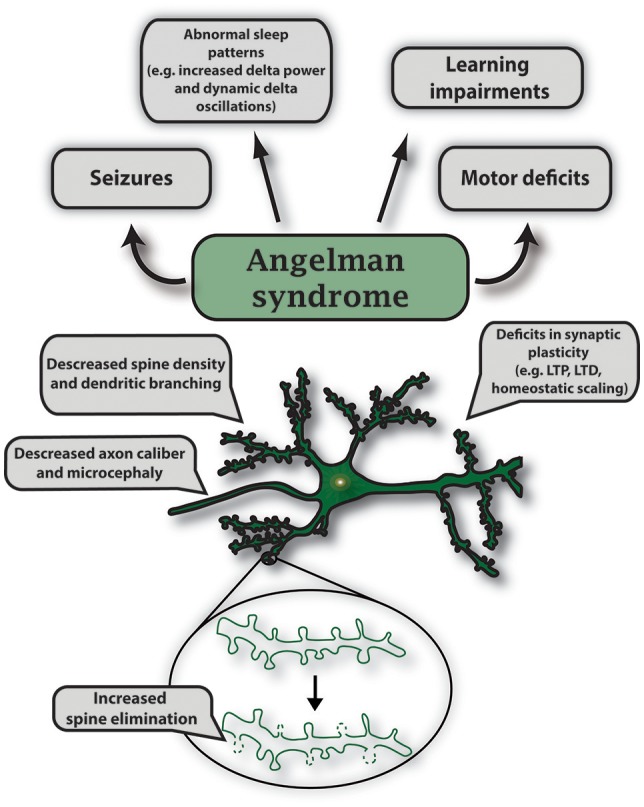
Characteristic features of gene mutations in *Ube3a* observed in Angelman syndrome. Angelman syndrome mouse models show abnormal sleep patterns that are accompanied by EEG recordings displaying increased delta power and dynamic delta oscillations; mouse strain-dependent seizures; learning impairments including intellectual disability and developmental delay. Specific symptoms vary in individual cases. In AS mouse models, maternal deletion of *Ube3a* causes microcephaly and leads to multiple deficits in synaptic plasticity such as decreased LTP induction, deficits in mGluR-dependent LTD and homeostatic scaling. Mutations in *UBE3A* alter neuronal morphology that includes decreased axon caliber and spine density, and increased spine elimination in select brain regions.

*UBE3A* encodes a HECT E3 ligase that is imprinted specifically in neurons of the central nervous system. Imprinting is established and maintained through expression of a long noncoding RNA on the paternal allele called the *UBE3A* antisense (*UBE3A-ATS*) transcript. As a result, only maternal UBE3A is expressed in neurons (Albrecht et al., 1997; Rougeulle et al., 1998; Runte et al., 2001, 2004; Yamasaki et al., 2003; Landers et al., 2004). Epigenetic regulation of *UBE3A* is similarly conserved in rodents, which has allowed researchers to generate AS murine models to study this disorder (Jiang et al., 1998). Using genetic engineering techniques, mouse models for AS were created to generate a viable maternally inherited *Ube3a* null mutation and a conditional reinstatement model has been created to restore the *Ube3a* null mutation during development (Jiang et al., 1998, 2010; Miura et al., 2002; Silva-Santos et al., 2015). AS mice display many AS-relevant phenotypes which include motor deficits, seizure susceptibility, learning impairments, and altered sleep homeostasis (Jiang et al., 1998, 2010; Miura et al., 2002; Cheron et al., 2005; Colas et al., 2005; van Woerden et al., 2007; Mulherkar and Jana, 2010; Ehlen et al., 2015). AS mice also exhibit deficits in multiple forms of synaptic plasticity such as long-term potentiation (LTP), long-term depression (LTD), metabotropic glutamate receptor (mGluR)-dependent LTD, homeostatic scaling, and ocular dominance plasticity (Figure 5; Jiang et al., 1998; Weeber et al., 2003; Dindot et al., 2008; Yashiro et al., 2009; Sato and Stryker, 2010; Pastuzyn and Shepherd, 2017).

The anatomical changes in AS have also been studied in great detail. In vertebrates, changes in dendritic spine dynamics are associated with alterations in learning, plasticity, and behavior throughout development (Dindot et al., 2008; Silva-Santos et al., 2015; Valluy et al., 2015; Pastuzyn and Shepherd, 2017). Notably, the density of dendritic spines was found to be reduced in a postmortem human AS patient using a traditional method of Golgi staining (Jay et al., 1991). Similarly, *Ube3a*^*m*−/*p*+^(AS) mice have a decrease in dendritic spines, an absence of normal induction of LTD and LTP in the visual cortex, and have defective ocular dominance plasticity (Dindot et al., 2008; Yashiro et al., 2009; Sato and Stryker, 2010). The use of two-photon microscopy has allowed researchers to increase the depth of imaging in living tissue and provide longitudinal changes in functional connectivity, cortical response, and neural activity with limited phototoxicity (Yang and Yuste, 2017). To observe changes in dendritic spine dynamics, AS mice were crossed with *Thy1*-GFP males (Feng et al., 2000; Kim et al., 2016). The resulting offspring of this cross allowed GFP expression in Layer 5 pyramidal neurons in both wild type (WT) and AS mice (Kim et al., 2016). Although dendritic spine density was not altered in AS mice, dendritic spine elimination was significantly increased during the end of the first month of postnatal life. However, enhanced spine elimination could be rescued when AS mice were deprived of visual experience by dark rearing (Kim et al., 2016).

Gross anatomical differences have been observed in multiple brain regions in the AS mouse model (Judson et al., 2017). AS mice exhibit microcephaly and have significant reductions in white matter tracts. These microstructural abnormalities were investigated using diffusion tensor imaging (DTI), a tool that provides unique information of the preferred orientation, myelination, and density in white matter specifically in axon bundles *in vivo* (Basser and Pierpaoli, 1996; Goodlett et al., 2009). Using electron microscopy, a decrease in axon caliber (reduced cross-sectional diameter) in myelinated axons was identified within the corpus callosum and sciatic nerves, which later revealed slower action potential rise kinetics compared to controls (Judson et al., 2017). Generally, microcephaly is linked to early neurological phenotypes such as hypotonia and seizures in infants (Fryburg et al., 1991). Therefore, the relationship between the deficits in postnatal brain growth and pathophysiology can be understood by examining the mechanism of this phenotype and the developmental consequences due to loss of *UBE3A*.

Optogenetics is another tool used to measure how neural populations affect the circuitry and function of the brain leading to behavioral phenotypes. This technique typically uses light to manipulate the activity of light-sensitive ion channels to spatially and temporally control cells in select brain regions (Klapoetke et al., 2014; Deisseroth, 2015; Yang and Yuste, 2017). Previously, a loss of UBE3A was found to enhance dopamine release in the mesoaccumbal pathway (Riday et al., 2012; Berrios et al., 2016). To evaluate the role of dopamine release in consummatory behavior, optogenetics was used to evaluate motivational behavior in a conditional AS model (*Ube3a*^*FLOX*/*p*+^) by crossing these mice to those that express CRE recombinase specifically in tyrosine hydroxylase neurons (*TH*^*CRE*^). These mice were then transduced with a CRE-dependent adeno-associated virus (AAV5)-channelrhodopsin-2 (H134R) fused to an enhanced yellow fluorescent protein (ChR2-eYFP) into the ventral tegmental area and an optical fiber was placed above the nucleus accumbens (Berrios et al., 2016). Mice were then trained on a specific schedule to nose-poke during optical stimulation. Mice with a loss of UBE3A in tyrosine-hydroxylase neurons demonstrated increased reward-seeking behavior via optical self-stimulation by suppressing the co-release of gamma-aminobutyric acid (GABA), an inhibitory neurotransmitter, in a non-canonical pathway (Berrios et al., 2016).

The generation of the UBE3A reinstatement model has allowed researchers to define neurodevelopmental windows that may rescue AS-related phenotypes. A conditional reinstatement mouse model of *Ube3a* was created using a CRE-dependent reinstatement of maternal *Ube3a* (*Ube3a*^*STOP*/*p*+^). Mice lacking maternal *Ube3a* displayed consistent impaired behavioral performance using a battery of behavioral tests (rotarod, marble burying, open field, nest building, forced swim test, and epilepsy) similar to the traditional AS mouse model (Silva-Santos et al., 2015). CRE-dependent reinstatement of UBE3A rescued motor deficits in adolescent mice. However, other AS behaviors such as anxiety, repetitive behavior, and epilepsy could only be rescued during early development (Silva-Santos et al., 2015).

Electrophysiology, specifically local field potential recordings, allow researchers to understand dynamic neural networks by measuring action potentials and graded potentials that reflect synaptic activity in the neural network (Herreras, 2016). Using this method, full recovery of hippocampal LTP was found at every time point of UBE3A reinstatement (Silva-Santos et al., 2015).

Using *in vivo* patch-clamp electrophysiology in the same mouse model above, AS mice were found to have increased excitability and reduced orientation tuning in regular-spiking GABA-ergic pyramidal neurons. However crossing *Ube3a*^*STOP*/*p*+^ with *Gad2-CRE* mice to specifically reinstate UBE3A in interneurons could rescue orientation tuning (Wallace et al., 2017).

A UBE3A reporter mouse was initially developed to assess regions in which UBE3A was imprinted (Dindot et al., 2008). The UBE3A-Yellow fluorescent protein knock-in mouse (*Ube3a*^*m*+/*pYFP*^) was used to identify compounds to unsilence the paternal *Ube3a* allele. The rationale behind this work was that the majority of AS individuals have a maternally inherited disruption of *UBE3A* unlike the paternal copy which is normal, but is not expressed due to epigenetic modifications (Lalande and Calciano, 2007). Using the *Ube3a*^*m*+/*pYFP*^ knock-in mouse in a high-content drug screen, topoisomerase inhibitors were found to unsilence the paternal *Ube3a* allele (Huang et al., 2012). Notably, the topoisomerase I inhibitor, topotecan, upregulated neuronal UBE3A expression in an AS mouse and downregulated *Ube3a-ATS* and *Snrpn* (*Small Nuclear Ribonucleoprotein Polypeptide N*) paternal gene expression in the brain *in vivo* (Huang et al., 2012). Another corresponding study also used *Ube3a*^*m*+/*pYFP*^ knock-in mice to test the efficiency of antisense oligonucleotides (ASOs) in depleting the *Ube3a-ATS* as another strategy to unsilence the paternal allele of *UBE3A* (Meng et al., 2015). The targeting of the paternal dormant allele provides a potential treatment option for AS by focusing on epigenetic modifications.

The development of AS patient-derived iPSCs has allowed researchers to study AS in a more human relevant context. (Chamberlain et al., 2010; Russo et al., 2015). These lines have been useful in studying the exact genomic disruption afflicted in AS patients and to understand human epigenetic *UBE3A* regulation to assist in identifying novel therapeutic strategies (Stanurova et al., 2016; Takahashi et al., 2017). AS-derived iPSCs have an increase in resting membrane potentials, decreased spontaneous synaptic currents, and a loss of LTP induction (Fink et al., 2017). These phenotypes could be rescued by treating AS-derived iPSCs with topotecan to unsilence the *UBE3A* paternal allele. Treatment with topotecan resulted in an increase in *UBE3A* mRNA expression which led to a shift to a more hyperpolarized resting membrane potential and restoration of action potential firing to control levels promoting normal neuronal excitability (Fink et al., 2017). Importantly, targeting the silenced paternal allele in AS patients might alleviate AS-related phenotypes such as intellectual impairments and developmental delays by increasing synaptic events (Fink et al., 2017). In another study, targeting CGI methylation *de novo* via introducing a CpG-free cassette into AS patient-derived iPSCs was able to correct abnormal DNA methylation and result in normal UBE3A expression in DA neurons (Takahashi et al., 2017).

In parallel to the use of transgenic mice and human iPSCs, clinicians have sought to discover indicators specific to AS patients. One of the methods used in human cases was electroencephalography (EEG) for which AS patients tend to display theta rhythmicity, epileptiform spike-wave changes, and increased delta rhythmicity (Vendrame et al., 2012). Delta power in the AS mouse and in AS children was compared. Results from these studies demonstrated an increase in delta power during wakefulness and sleep in both AS mice and children with AS compared to matched controls (Sidorov et al., 2017). These studies reveal, that in AS, loss of UBE3A results in large-scale disruptions in rhythmic neural activity and shows that EEGs may serve as a potential biomarker for not just AS, but for other RNDs that have seizure phenotypes.

It is worth noting duplication of the 15q11-q13 region of the maternal chromosome harboring the *UBE3A* gene is also a common and highly penetrant factor of autism spectrum disorder (ASD) pathogenesis (Figure 1 and Supplementary Table 1), and an increased dosage of the *UBE3A* gene is associated with developmental delay and neuropsychiatric phenotypes (Cook et al., 1997; Glessner et al., 2009; Noor et al., 2015). *UBE3A* can function as both an E3 ligase and a transcriptional co-activator (Scheffner et al., 1993; Dindot et al., 2008). It is expressed monoallelically in neurons and is involved in many previously stated functions such as maintaining the proper level of dendritic branching, synapse formation, and controlling the frequency of mEPSCs (Lu et al., 2009; Greer et al., 2010; Margolis et al., 2010; Khatri et al., 2017). Because previous research has parsed out the importance of expression sensitivity of UBE3A in two disorders, developing drugs that can control the expression of either the paternal or maternal allele or those that modulate neuronal activity would be beneficial to this patient population. Moreover, due to the emergence of early symptoms in AS and ASD, individuals with *UBE3A* disruptions can be monitored throughout their lifetime to prevent or alleviate ongoing symptoms such as seizures or ataxia.

### Other rare neurological disorders

Mutation in E3 ligase genes are associated with a vast multitude of RNDs (Figure 3 and Supplementary Table 2). For the majority of RNDs, there is not strong supporting evidence to indicate a causal link between them. Many of the RNDs mentioned in detail below have implied a possible relationship between the RND and an E3 ligase through familial case studies by looking at probable critical regions on chromosomes to screen out less important genes (Sekine and Makino, 2017).

#### Epilepsy

Disorders that encompass seizure-like phenotypes have links to neurodegeneration (Wong, 2013; Dingledine et al., 2014). Epilepsy is a neurological disorder involving a long-term susceptibility to seizures caused by atypical neuronal activity in the brain (Fisher et al., 2005). About 3.4 million people, both adults and children, in the U.S. have active epilepsy (Zach and Kobau, 2017). Although there are many classifications of epileptic disorders, for the purpose of this review, we will only focus on specific rare epileptic types associated with E3 ligases.

Infantile spasms (IS) are characterized by the onset of seizures that occur in clusters during the first year of life. IS patients display irregular EEG readings known as hypsarrhythmia that is thought to cause developmental dysfunction (Lux and Osborne, 2004). The incidence of IS occurs in about 0.025–0.05% of live births (Taghdiri and Nemati, 2014). IS is caused by a deletion in the chromosome region 1p36. This region was identified using fluorescence *in situ* hybridization (FISH), a cytogenetic technique that uses fluorescence microscopy to visualize fluorescent probes designed to detect complementary nucleic acid sequences on chromosomes (Ratan et al., 2017). The fluorescent probe is RNA or single-stranded DNA labeled with fluorophores through nick translation or PCR that hybridize to its target with antibodies or biotin (Levsky and Singer, 2003). In human case studies, one subject was identified to have a chromosome deletion with copy number variation in the *KLHL17* (Kelch-like family member 17) gene (Figure 3 and Supplementary Table 2). This gene encodes an E3 ligase that is thought to play a role in actin-based neuronal function (Paciorkowski et al., 2011). In this study, the use of FISH was helpful in screening E3 ligases selectively to epileptic disorders. Even so, more studies would need to prove that there is a more critical region for IS that includes *KLHL17* and to confirm that *KLHL17* is a causative gene for IS.

Adult myoclonic epilepsy (AME) is associated with myoclonic jerks and twitches as well as finger shaking movement. Worldwide prevalence of AMEs remains unknown, but there are geographic variations of different genes associated with this disorder (Delgado-Escueta et al., 2003). AME is linked to missense mutations in the HECT E3 ligase, *UBR5* (Figure 3 and Supplementary Table 2; Kato et al., 2012). *UBR5* was identified using whole exome enrichment and sequencing (WES) via NGS by using RNA probes to find single nucleotide variants (SNVs) and single nucleotide polymorphisms (SNPs) (Chen et al., 2015). While NGS is a type of technology that allows for high throughput sequencing, WES is a type of NGS that is more focused on sequencing protein-coding regions of a genome that contain mutations (Seleman et al., 2017). *UBR5* mutations were identified in affected family members with AME but not in unaffected groups or unaffected family members (Kato et al., 2012). UBR5 has many functional roles including maturation and transcriptional regulation of mRNA, cell cycle, extraembryonic development, tumor suppression and regulation of the DNA topoisomerase II binding protein (TDP2). Other functions include suppression of another E3 ligase, RNF168, in response to DNA damage and prevention of growth of ubiquitinated chromatin in response to chromosomal damage (Gudjonsson et al., 2012). Similar to IS, further studies are needed to verify the importance of mutations in the *UBR5* gene and its association with AME. Researchers have only begun to scratch the surface of finding altered E3 ligase functions in epilepsy making it difficult to currently manage epileptic patients with these specific gene mutations due to limited research in understanding the role of ubiquitinated proteins in epilepsy. Along with further investigation of E3 ligases, animal models with knock-out genes such as *KLHL17* or *UBR5* or even knock-in mouse models would be beneficial in determining the functionality of these genes for both *in vitro* and *in vivo* experiments. The isolation of iPSC cells from individuals with epilepsy and brain imaging tools such as EEGs might be useful to identify biomarkers.

#### Gordon holmes syndrome

Gordon Holmes syndrome (GHS) is another RND that has recently gained more attention. The clinical symptoms include ataxia and hypogonadotropic hypogonadism, cognitive impairment, dysarthria, cerebellar ataxia, and in some cases dementia (Haines et al., 2007; Margolin et al., 2013; Alqwaifly and Bohlega, 2016). GHS is part of a subset of disorders called autosomal recessive hereditary cerebellar ataxias (ARCA) with extracerebellar symptoms such as dementia (Heimdal et al., 2014). The prevalence of GHS remains unknown. Whole exome sequencing studies have established that homozygous mutations in the E3 ligase STIP1 homology and U-Box containing protein 1 (*STUB1*) also known as carboxy terminus of Hsp70-interacting protein (*CHIP*), results in ataxia and hypogonadism with a frequency of 2.3% in GHS patients (Shi et al., 2014; Hayer et al., 2017; Figure 3 and Supplementary Table 2). Evidence in clinical familial cases have also demonstrated that mutations in *STUB1/CHIP* were identified in patients with ARCA along with cognitive impairment (Heimdal et al., 2014). Functionally, *STUB1/CHIP* is a gene that encodes the protein CHIP which is a U-box dependent E3 ligase involved in chaperoning proteins (Jiang et al., 2001). In assessing neurological behaviors, *Chip* knockout mice have decreased motor, sensory and cognitive function. These impairments were also associated with abnormal cellular morphology of Purkinje cells and other cortical cell layers resulting in cerebellar dysfunction (Shi et al., 2014).

Markedly, GHS has been associated with both missense and nonsense mutations in the RBR E3 ligase, *RNF216/TRIAD3* (Margolin et al., 2013) (Figure 3 and Supplementary Table 2). In familial genetic studies, patients diagnosed with ataxia and hypogonadotropic hypogonadism had compound heterozygous mutations in *RNF216* whose variants were predicted to be deleterious compared to controls (Margolin et al., 2013). This implicated *RNF216* as a causative gene for this disorder (Margolin et al., 2013). In one deceased patient, histopathological examination revealed atrophy of the cerebellum, gliosis, loss of inferior olivary neurons and cerebellar Purkinje cells, and loss of neurons in hippocampal regions CA3 and CA4. Moreover, ubiquitin-immunoreactive nuclear inclusions were found in the CA1, CA2, and dentate gyrus of the hippocampus further providing an anatomical basis for dementia. Longitudinal studies of the clinical symptoms of GHS patients identified dysarthria in early stages of life, while ataxia and dementia developed later on in adulthood indicated by neuroimaging results that revealed cerebellar and cortical atrophy (Margolin et al., 2013).

Considering hypogonadotropic hypogonadism is another feature of GHS, the endocrine system in GHS individuals was examined. Decreased levels of luteinizing hormone (LH) and pituitary dysfunction were detected indicating gonadotropin-releasing hormone (GnRH) secretion deficiencies; indeed, when robust pulses of GnRH were administered, gonadotropin levels and reproductive function were restored (Margolin et al., 2013). Another clinical case study showed cerebellar and cortical atrophy through the use of fMRIs and fluid-attenuated inversion recovery (FLAIR) brain imaging in two patients with homozygous mutations of a splice variant of *RNF216* (Alqwaifly and Bohlega, 2016). FLAIR is a MRI technique that contrasts the tissue T2 prolongation and the cerebrospinal fluid signal so that lesions near the cerebrospinal fluid are revealed (Saranathan et al., 2017). These patients also confirmed low levels of LH and additionally testosterone, but testosterone treatment normalized secondary sexual characteristics (Alqwaifly and Bohlega, 2016). A recent case study identified additional mutations in *RNF216* in a patient with GHS who had progressive cognitive decline that correlated with high signal intensity within the white matter of both cerebral hemispheres with gray matter lesions in the thalami, cerebellar atrophy, and high T2 signals in the midbrain (Mehmood et al., 2017). These clinical case studies support the strong relationship between behavioral phenotypes and their corresponding biological insults.

As opposed to using mouse models to support the role of RNF216 in GHS, zebrafish were used to test the functionality of the gene by injecting morpholino oligonucleotides (MO) in order to silence *rnf216*. This resulted in decreased size of the eye cup, optic tecta, and head size along with disorganization of the cerebellum. These phenotypes were rescued with co-injection of human *RNF216* mRNA (Margolin et al., 2013). Complementing these data with transgenic mouse lines would provide a strong foundation to support the genetic studies of familial variability both *in vitro* and *in vivo*.

*RNF216* encodes multiple RING finger E3 ligase isoforms (TRIAD3A-TRIAD3E) and plays a major role in inflammation (Chen et al., 2002; Chuang and Ulevitch, 2004; Fearns et al., 2006; Nakhaei et al., 2009; Shahjahan Miah et al., 2011; Xu et al., 2014). The first neuronal function for *RNF216* was the identification of the immediate early gene, activity-regulated cytoskeletal-associated protein (Arc), as a substrate of TRIAD3A. TRIAD3A was found to directly ubiquitinate Arc and mediate its turnover by the UPS in mouse primary neurons (Mabb et al., 2014). Using a technique called total internal reflection fluorescence microscopy (TIRFM), TRIAD3A was also found to localize at clathrin-coated pits resulting in altered trafficking of α-amino-3-hydroxy-5-methyl-4-isoxazolepropionic acid (AMPA) receptors, principal excitatory receptors that mediate the majority of fast excitatory synaptic transmission in the nervous system (Mabb et al., 2014). Using shRNA to reduce levels of TRIAD3A, Arc-dependent forms of synaptic plasticity were found to be altered most likely due to disruptions in AMPA receptor trafficking (Mabb et al., 2014). Additionally, viral transduction of *Triad3* shRNA in the hippocampus of mice led to deficits in learning in the Morris water maze, a spatial-dependent learning task (Husain et al., 2017).

Due to *STUB1/CHIP*'s role in directing chaperone proteins for proteasomal degradation, patients with this mutation could partake in treatment options that target these substrates to promote the growth and maintenance of Purkinje cells and other cortical cell layers. Regarding *RNF216*, particularly TRIAD3A, therapeutic inventions to induce Arc-dependent forms of synaptic plasticity could possibly prevent or at least delay the dementia phenotype. In conjunction, targeting substrates of the inflammatory pathway could prevent abnormal cell death. Research to elucidate the roles of *RNF216* isoforms would be helpful in creating viable treatment options for patients. The generation of additional transgenic animal models and the use of iPSCs would be beneficial in determining the effects of both *STUB1* and *RNF216* disruptions on a cellular level and would aid in establishing additional substrates and pathways leading to behavioral phenotypes.

#### Louis bar syndrome

Louis Bar syndrome, commonly referred to as ataxia-telangiectasia (AT), is identified by its symptoms of ataxia, telangiectasia, elevated alpha-fetoprotein, microcephaly, pulmonary failure, radiosensitivity, immunodeficiency, dysmorphic features, and learning difficulties with a prevalence of 0.001–0.0025% in live births (Boder and Sedgwick, 1970; Swift et al., 1986; Richard and Susan, 1999). Clinical studies showed an occurrence of homozygous nonsense mutations in *RNF168* in patients with AT and radiosensitivity (Figure 3 and Supplementary Table 2). In addition, when screening for irradiation-induced nuclear foci containing 53BP1, AT lymphoblastoid cells showed a deficiency in the *RNF168* pathway (Devgan et al., 2011). *In vitro* studies using shRNA showed that knock-down of *RNF168, RNF8* and *53BP1* in a CH12F3-2 mouse B cell line resulted in a significant reduction in class-switch recombination (CSR) (Ramachandran et al., 2010). *RNF168* plays a pivotal role following DNA double strand breaks (DSBs). During DNA damage, RNF168 is recruited to H2A-type histones and amplifies H2A lysine 63-linked ubiquitin conjugates mediated by another E3 ligase RNF8 (Stewart et al., 2009). This results in the accumulation of 53BP1, a protein important for double-stranded break repair, and BRCA1, a tumor suppressor protein, that are recruited to the sites of DNA damage critical for mediating cell cycle checkpoints and DNA repair (Stewart et al., 2009). While studying the immunology and radiological aspects of Louis-Bar syndrome is valuable, a nice correlate would be to determine if mutations in *RNF168* causes other symptoms such as ataxia, telangiectasia or pulmonary failure. This would involve the use of transgenic animal models to study behavioral phenotypes. It would also be conducive to give attention to knock-out or overexpression of *RNF168* in other cell types such as neurons or glial cells to determine if mutations in these cell types may result in impaired behavioral phenotypes.

#### Moyamoya disease

Moyamoya Disease (MMD) is an RND that shows unique symptoms of steno-occlusion of the terminal side of the internal carotid artery causing abnormal vascular networks at the base of the brain (Ma et al., 2016). This disease has recently gained attention because it is thought to be a causative factor of stroke in both adults and children (Veeravagu et al., 2008). Although the progression of pathogenesis and prevalence is still unclear, missense mutations in *RNF213* have been identified in 95% of familial cases and 73% of sporadic clinical cases (Kamada et al., 2011) (Figure 3 and Supplementary Table 2). Among the 30 *RNF213* variants listed from the Human Gene Mutation Database (HGMD), R4810K is the only variant that is strongly associated with MMD (Jang et al., 2017; Supplementary Table 2). A familial clinical case showed one patient with the R4810K mutation did not show any abnormalities with neuroimaging tools during childhood, but was diagnosed with MMD 10 years later after showing symptoms (Aoyama et al., 2017). This suggests that determining the time course of disease progression is an important factor in diagnosis and treatment.

The role of *RNF213* in MMD was examined by subjecting WT mice to transient middle cerebral artery occlusion (tMCAO) and measuring mRNA expression of *RNF213* both by *in situ* hybridization and RT-PCR, finding that *RNF213* was upregulated compared to controls and its expression was predominantly in neurons (Sato-Maeda et al., 2016). A MMD mouse model was created to produce homozygous recessive *RNF213* (*RNF213*^−/−^) animals whose cervical and cranial arteries were examined using magnetic resonance angiography (MRA) (Sonobe et al., 2014). Although there was no difference in MRA readings between WT and transgenic mice, common carotid artery ligation which induced vascular hyperplasia, resulted in thinner intima and medial layers in *RNF213*^−/−^ mice compared to WT controls that exhibited hyperplasia (Sonobe et al., 2014). This supports a role of *RNF213* in brain ischemia, a symptom of MMD, but further studies are needed to understand the mechanism of RNF213 action in MMD. Notably, iPSCs and iPSC-derived vascular endothelial cells (iPESCs) were taken from MMD patients that had reduced angiogenic activity. Microarrays confirmed that many mitotic-phase associated genes and securin, an inducer of angiogenesis and inhibitor of premature sister chromatin separation, were downregulated with this *RNF213* genotype (Hitomi et al., 2013). RNAi-mediated depletion of securin also impaired tube formation without affecting proliferation of iPSCs (Hitomi et al., 2013). Using iPSCs can be useful as an *in vitro* model to study not only the pathophysiology of *RNF213*, but also to investigate specific drug targets for therapeutic intervention.

#### Juberg-Marsidi syndrome

Juberg-Marsidi syndrome is a rare congenital X-linked disorder that specifically affects males. Symptoms consist of mental retardation, delay in developmental milestones, muscle weakness, hypotonia, growth retardation, deafness, microgenitalism, microcephaly, and additional physical abnormalities (Villard et al., 1996). The prevalence remains unknown. Using NGS, Juberg-Marsidi syndrome was found to be associated with mutations in the HECT E3 ubiquitin ligase, *HUWE*1, implicating it as a possible candidate gene for this X-linked disorder (Nava et al., 2012; Friez et al., 2016; Figure 3 and Supplementary Table 2). In evaluating the function of HUWE1 and its substrates, a conditional knock-out mouse model was generated to delete the HECT domain of HUWE1. Mice were crossed with *GFAP-CRE* deleter mice to specifically target *HUWE1* deletion in cerebellar granule neuron precursors (CGNPs) and radial glia (D'Arca et al., 2010). These mice were found to have high lethality around postnatal day 21 and cerebellar abnormalities including defects in cell cycle exit and granule cell differentiation with an ataxic phenotype caused by uncontrolled proliferation of CNGPs. This was associated with an increase in the abundance of a *HUWE1* substrate, N-Myc (D'Arca et al., 2010). There are many different functions of HUWE1 including regulating neural differentiation and proliferation via catalyzing the polyubiquination and degradation of *MYCN*, which encodes the N-myc oncoprotein; ubiquitination of the tumor suppressor protein, p53; and regulation of *CDC6* levels, essential for DNA replication, after DNA damage (Yoon et al., 2004; Hall et al., 2007; Zhao et al., 2008). In parallel with the work mentioned above, it would be favorable to determine how the overexpression of HUWE1 alters differentiation of the cerebellum. Indeed, disruption of HUWE1 in human iPSCs would assist in establishing critical developmental time points related to postnatal lethality.

#### Opitz G/BBB syndrome

The Opitz G/BBB syndrome (OS) is another congenital disorder that involves two forms: X-linked and autosomal dominant found on chromosome 22. Both forms have similar abnormalities due to defects of the midline structures which include growth delay, microcephaly, polydactyly, cleft palate, mental retardation, seizures, heart defects, hypertelorism, and deafness. (Robin et al., 1996). The prevalence of this disease is unknown. In particular, the X-linked form is caused by a mutation in the *MID1* gene, an E3 ligase that is a member of the B-box family of zinc finger proteins with a RING-finger motif involved in anchoring proteins to microtubules (Quaderi et al., 1997; Figure 3 and Supplementary Table 2). *In vivo* studies using a *Mid1*-null mouse line demonstrated OS phenotypes observed in affected humans. For example, prenatal cerebellar defects lead to dysfunction of primitive fissures and definitive boundaries resulting in motor discoordination and motor learning deficiencies (Lancioni et al., 2010). This work provides insight into the genetic causes underlying the behavior observed in OS. The use of primary neuron cultures and stem cells to understand how differentiation of the midline is affected and elucidation of the pathway the underlies this mechanism would be informative in understanding the origin leading to these OS symptomologies.

## Conclusions

The current studies on E3 ligases and their implication in neurological disorders is still an open field where research using diverse, emerging technologies would benefit. Molecular diagnosis of neurological disorders requires accurate, efficient, and cost-effective methods. Traditionally, standard PCR was helpful in detecting short sequences of repeat expansions. The emergence of Sanger sequencing allowed for sequencing of the entire human genome but was considerably time-consuming and cost prohibitive (Goldfeder et al., 2017). The studies discussed above show the benefits of using rapid and cost-effective NGS platforms in identifying gene variants and novel disease genes. Narrowing down the genetic causes of neurological diseases will allow clinicians and health care professionals to advise and administer specialized treatments at appropriate times to assist in the reduction of disease burden over time.

The prevalence of E3 ligase disruptions in such a broad array of neurological diseases suggests disruption in ubiquitin pathways may be a major driving force. The abundance of E3 ligase genes mutated in neurological disease (Supplementary Tables 1, 2) indicates that targeting the ubiquitin pathway might have utility for a range of neurological disorders. However, this serves as a great challenge to researchers given our lack of a comprehensive understanding of E3 ligases and their role in neurodevelopment, neuronal maintenance, and a lack of information of E3 ligase substrates. This is further clouded in difficulties in developing intervention therapies due to the diversity and complexity of the ubiquitin pathway (Huang and Dixit, 2016). It is worth noting that for the few drugs that have been developed to target the ubiquitin pathway, most are meant to inhibit or disrupt function. Considering many of the E3 ligases mutated in neurological disease are related to a loss of enzyme function, inhibitors targeting these enzymes would not be beneficial (Bondeson et al., 2015; Galdeano, 2017). However, the finding that there are multiple E3 ligases that are disrupted in similar disease subsets (e.g., ASD) indicates a potential nexus of biological and functional convergence (Figures 1, 3). Intriguingly, with the exception of GHS, we found that RNDs appear to be associated with one type of E3 ligase domain class (Figure 4), whereas CNDs tend to share E3 ligase domain classes (Figure 2).

The emergence of methods such as hydrophobic tagging might be useful for the targeting of E3 ligase substrates that undergo protein degradation (Neklesa et al., 2011; Huang and Dixit, 2016) but this requires the identification of substrates. Moreover, E3 ligase substrates may undergo ubiquitination that does not lead to subsequent degradation by the UPS. In order to maximize the potential for therapeutic treatments especially for RNDs, future studies that answer the following questions are warranted: What is the full list of mutated genes that encode for E3 ligases in neurological disease? NGS and access to patient populations for RNDs and CNDs would assist in this endeavor. What is the full range of substrates that are targeted by disease-associated E3 ligases? One of the most extensive lists exists for the E3 ligase Parkin but other E3 ligase substrates remain elusive (Panicker et al., 2017). What are the functions of the E3 ligases that are disrupted in neurological disorders? Note that for many RNDs, E3 ligase function in the nervous system is undefined. Are E3 ligases that are mutated in similar disorders have overlapping biological functions? Very little is known about how these enzymes function in the nervous system. Finally, for the ever-growing list of CNDs and RNDs that exhibit symptomatic heterogeneity, how does one select drug targets and develop viable therapeutic treatments? This, of course, will be the greatest of challenges for researchers.

## Author contributions

YH and AC: generated the list of E3 ligases implicated in neurological disorders found in Supplementary Tables 1, 2. AG and AM: Validated the list of E3 ligases and wrote the manuscript; AG and YZ: Created the figures.

### Conflict of interest statement

The authors declare that the research was conducted in the absence of any commercial or financial relationships that could be construed as a potential conflict of interest.

## Supplementary material

Supplementary Table 1 List of E3 ubiquitin ligases mutated in common neurological disorders (CNDs).

Supplementary Table 2 List of E3 ubiquitin ligases mutated in rare neurological disorders (RNDs).

## Glossary

**ataxia**: inability to coordinate voluntary muscle movements due to dysfunction of the central nervous system, not due to muscle weakness **bradykinesia:** extreme slowness in movements and reflexes

**dysarthria**: slurred speech due to dysfunction of the central nervous system

**dystonia**: abnormality of movement and muscle tone

**gliosis**: abnormal production of glia cells

**homeostatic scaling:** form of synaptic plasticity in which single neurons can regulate their own excitability in relation to network activity

**hyperplasia**: abnormal production of cells in tissues

**hyperreflexia**: overactivity of physiological reflexes

**hypertelorism**: abnormal increase in distance between two body parts (e.g. between the eyes)

**hypotonia**: having deficient muscle tone

**hypogonadotropic hypogonadism**: decreased activity of the gonads (testes and ovaries) due to deficiency of gonadotropins (LH and FSH) and diminished levels of sex steroid hormones

**hypsarrhythmia**: abnormal encephalogram characterized by disorganized arrangement of spikes

**long term depression (LTD)**: a form of activity-dependent plasticity that weakens a specific set of synapses due to a patterned stimulus that reduces the excitatory postsynaptic potential

**long term potentiation (LTP)**: a form of activity-dependent plasticity that persistently strengthens a set of synapses due to a patterned stimulus

**microcephaly**: abnormal smallness in circumference of the head that occurs at birth or within the first few years of life

**myoclonic jerk:** brief, involuntary twitching due to rapid contraction and relaxation of muscles

**nuclear inclusion:** clusters of a substance (e.g. proteins) that occur in the nucleus of a cell

**ocular dominance plasticity:** stripes of neurons located in the visual cortex that span many cortical layers that preferentially respond from one eye or another and are modified by activity-dependent changes in neuronal connections during a critical period

**telangiectasia**: abnormal dilation of the subcutaneous vascular system

**radiosensitivity**: quality of being quick to respond to slight changes in radiant energy

**steno-occulsion**: proximal narrowing or blockage of a blood vessel

**telangiectasia**: threadlike red patterns on the skin caused by widened venules
